# Clinical evaluation of novel deep learning‐based auto‐segmentation software: Utility and potential pitfalls

**DOI:** 10.1002/acm2.70757

**Published:** 2026-08-25

**Authors:** Ryota Tozuka, Masahide Saito, Masaki Matsuda, Tomoko Akita, Hikaru Nemoto, Zhe Chen, Takafumi Komiyama, Kazuma Mochizuki, Noriyuki Kadoya, Keiichi Jingu, Hiroshi Onishi

**Affiliations:** ^1^ Department of Therapeutic Radiology University of Yamanashi Chuo Yamanashi Japan; ^2^ Department of Radiation Oncology Tohoku University Graduate School of Medicine Sendai Miyagi Japan

**Keywords:** artificial intelligence, auto segmentation, clinical evaluation, deep learning, organ‐at‐risk, radiotherapy

## Abstract

**Background:**

Accurate contouring of target volumes and organs at risk is critical in radiotherapy. While deep learning (DL) models offer automated contouring, their clinical applicability to real‐world cases containing anatomical variations and artifacts requires rigorous validation.

**Purpose:**

To evaluate the clinical accuracy and potential vulnerabilities of RatoGuide, novel DL‐based auto‐segmentation software, using a dataset including atypical cases derived from routine clinical practice.

**Methods:**

This single‐center retrospective study included 69 thoracic and male pelvic cases. The cohort was intentionally selected to encompass diverse anatomies and artifacts (e.g., pacemakers, SpaceOAR implants, artificial femoral head replacements, and unilateral atelectasis). Auto‐contours generated by RatoGuide were compared with expert‐approved manual contours. Performance was evaluated quantitatively using the Dice Similarity Coefficient (DSC) and 95th percentile Hausdorff Distance (HD95), and qualitatively via a 5‐point visual assessment scale by four independent reviewers. Statistical comparisons between cohorts were performed using the Mann‐Whitney U test. Additionally, a dosimetric evaluation was conducted for male pelvic cases to assess clinical impact.

**Results:**

In typical cases, the software maintained high segmentation accuracy (thorax: mean DSC 0.856, mean HD95 6.89 mm; male pelvis: mean DSC 0.874, mean HD95 4.11 mm). However, performance declined in atypical cohorts (thorax: mean DSC 0.808, *p =* 0.0457, mean HD95 12.22 mm, *p =* 0.0002; male pelvis: mean DSC 0.828, *p =* 0.1620, mean HD95 6.16 mm, *p =* 0.0075). Notable decreases in accuracy were observed in challenging scenarios, such as artificial femoral head replacements (DSC: 0.754) and unilateral atelectasis (DSC: 0.784). Qualitative assessment revealed that errors were primarily due to anatomical factors and artifacts. Furthermore, the dosimetric evaluation identified one critical false‐negative error where a dose constraint violation was overlooked when the DL contour was used.

**Conclusions:**

RatoGuide demonstrated favorable performance in typical cases, but accuracy declined in atypical cases with artifacts or altered anatomy. For clinical implementation, rigorous visual verification and manual review by experts are essential, particularly for atypical cases and organs in high‐dose gradient regions.

## 1 INTRODUCTION

Accurate contouring of target volumes and organs at risk (OARs) is a critical prerequisite for optimizing treatment plans and evaluating dose distributions in radiation therapy. However, the manual nature of this process, typically conducted by radiation oncologists and medical physicists, is not only time‐consuming but also suffers from significant inter‐observer variability.^1^, ^2^ These limitations can adversely affect tumor control and increase the probability of normal tissue toxicity.^3^, ^4^

Artificial intelligence (AI), particularly deep learning (DL), offers an effective solution to address these challenges and is increasingly used for the auto‐segmentation of target volumes and OARs.^5^, ^6^, ^7^ Several studies have demonstrated that AI‐assisted segmentation can reduce the clinical workload and decrease both intra‐ and inter‐observer variability.^8^, ^9^, ^10^, ^11^ In fact, multiple commercial DL‐based auto‐segmentation software packages are now available and have become integral to modern, high‐precision radiotherapy workflows.^12^, ^13^, ^14^, ^15^, ^16^, ^17^, ^18^, ^19^

However, the importance of quality assurance (QA) for DL‐based auto‐segmentation has been emphasized given that segmentation failures can occur.^20^, ^21^ Notably, DL models trained on curated datasets may show reduced performance when applied to real‐world clinical cases with anatomical variations or minor artifacts. Although many reports have evaluated commercial auto‐segmentation software packages, they have primarily focused on overall geometric indices without anatomical variations.^12^, ^17^, ^19^ While some studies have evaluated clinical acceptability through visual or dosimetric assessments, there remains a need for more comprehensive investigations that simultaneously compare typical and atypical cases using these multifaceted approaches.^22^, ^23^, ^24^, ^25^ Therefore, verifying the applicability to actual clinical workflows through such comprehensive approaches is a critical step for software validation.^16^, ^21^, ^26^, ^27^, ^28^, ^29^

RatoGuide (AiRato, Inc., Sendai, Japan) is novel radiotherapy treatment planning support software that features both auto‐segmentation and automated planning capabilities. Although several reports have evaluated its automated planning functions, its auto‐segmentation performance has not yet been sufficiently assessed.^30^, ^31^, ^32^, ^33^ To our knowledge, only one study has evaluated RatoGuide's auto‐segmentation, which was limited to the male pelvic region and relied solely on geometric metrics.^34^ To address these gaps, we investigated the clinical applicability of RatoGuide across both thoracic and pelvic regions. In this study, we comprehensively evaluated its performance by combining geometric metrics, stratified comparisons of typical and atypical clinical cases, expert visual assessments to classify specific failure modes, and partial dosimetric evaluations, thereby elucidating its practical utility and limitations in routine clinical workflows.

## 2 MATERIALS AND METHODS

### 2.1 Patient dataset and image acquisition

This study was approved by the institutional review board of the University of Yamanashi. The approval number was CS0010. This retrospective study included 69 patients treated at our institution between November 2018 and March 2025. To evaluate the software's performance in routine clinical practice, cases involving the thorax and male pelvic regions were selected from our database. The organs evaluated in the thorax region included the left and right lungs, spinal canal, esophagus, heart, aorta, pulmonary artery, and superior vena cava. In the male pelvis region, the evaluated organs comprised the prostate, urinary bladder, rectum, seminal vesicles, and left and right femoral heads. The cohort was purposely selected to encompass diverse tumor types and anatomical variations to identify potential failure modes in real‐world scenarios. In this study, cases were classified into “typical” and “atypical” cohorts based on the presence or absence of significant anatomical deformations or imaging artifacts. Patient characteristics are summarized in Table 1. For the thoracic region, the “typical” cohort included standard early‐stage (T1) and advanced‐stage (T3) primary lung cancer cases (staged according to the UICC 8th edition; detailed histological types are summarized in Table 1), whereas the “atypical” cohort comprised thoracic cases not limited to primary lung cancer, with a pacemaker or unilateral atelectasis. For the male pelvic region, the “typical” cohort consisted of standard anatomy prostate cancer cases, while the “atypical” cohort included cases with specific challenges: SpaceOAR implants, excessive rectal gas, and artificial femoral head replacements. Planning CT scans were acquired using an Aquilion LB (Canon Medical Systems, Otawara, Japan). All patients underwent non‐contrast planning CT scanning with the following parameters: 120 kV, 250 mA, and 2‐mm slice thickness. Metal artifact reduction was applied in selected cases. Necessary pre‐treatment procedures, such as bladder filling for prostate cancer irradiation, were performed as clinically indicated.

**TABLE 1:** Clinical characteristics of the cases included in this study

Region		Details	Number of patients	Remarks
Thorax	Typical	Early‐stage (T1)	10	AC (n=1), SqCC (n=6), CDLC (n=3)
		Advanced‐stage (T3)	10	AC (n=2), SqCC (n=3), SCLC (n=2), CDLC (n=3)
	Atypical	With a pacemaker	5	
		Unilateral atelectasis	10	
Male Pelvis	Typical	Standard anatomy	10	
	Atypical	SpaceOAR implant	10	
		Excessive rectal gas	9	
		Artificial femoral head replacement	5	

**Abbreviations**: AC, adenocarcinoma; CDLC, clinically diagnosed lung cancer; SCLC, small cell lung cancer; SqCC, squamous cell carcinoma.

The clinical contours used for treatment served as the ground truth (GT) for this study. Delineation was performed on either the Pinnacle (Philips Radiation Oncology Systems, Fitchburg, WI, USA) or RayStation (RaySearch Laboratories, Stockholm, Sweden) treatment planning systems, based on standard delineation guidelines.^35^, ^36^, ^37^, ^38^, ^39^ Where appropriate, fused MRI or contrast‐enhanced CT images were used to assist in accurate delineation. Initially drawn by a radiation oncologist or a medical physicist, all contours were subsequently reviewed, revised if necessary, and formally approved by a senior radiation oncologist with more than 7 years of experience.

To ensure fair evaluation for longitudinally extensive organs (e.g., the esophagus and spinal canal), the craniocaudal extent of the AI contours was adjusted. Since the GT is often limited to the clinically relevant region, AI contours extending beyond the GT were cropped to match the GT limits in the superior‐inferior direction. Conversely, if the AI contour was shorter than the GT, no adjustment was made to account for under‐segmentation.^40^

### 2.2 Generation of DL‐based contours

RatoGuide (Version 1.6.1.7, research version) is DL‐based automatic segmentation software utilizing the UNesT model architecture.^41^ Specific details regarding the training dataset and pre‐processing algorithms are proprietary to the manufacturer and were not disclosed.

In the experimental workflow, planning CT datasets in DICOM format were imported into RatoGuide using external storage devices, as the system operated in a standalone environment isolated from the institutional network. Upon selecting the target structures, the auto‐segmentation process was executed. The generated contours were subsequently exported as DICOM RT Structure sets to RayStation for evaluation.

### 2.3 Quantitative and qualitative evaluations

To evaluate the auto‐contours generated by RatoGuide, we conducted both quantitative and qualitative assessments.

For the quantitative evaluation, the Dice Similarity Coefficient (DSC) and the 95th percentile Hausdorff Distance (HD95) were employed. The DSC is a metric that indicates the degree of overlap between two segmented volumes, A and B, and is defined by the following equation:

$$DSC=\frac{2\times \left|A\cap B\right|}{\left|A\right|+\left|B\right|}$$

where |A| and |B| represent the volumes of the two segments. Generally, a DSC value of 0.8 or higher is considered to indicate good agreement.^42^

Furthermore, the HD95 was utilized to evaluate the boundary accuracy. The Hausdorff Distance measures the maximum distance from a point in one set to the nearest point in the other. To eliminate the impact of outliers, the 95th percentile of these distances is used instead of the maximum. It is defined based on the directed Hausdorff distance h(A,B):

$$HD\;\left(A,\;B\right)=max\left(h\left(A,B\right),\;h\left(B,A\right)\right)$$

where $h\left(A,\;B\right)=max_{a\in A}min_{b\in B}∥a-b∥$. For HD95, the 95th percentile is calculated in place of the maximum operation.

While DSC and HD95 are widely used metrics, they have known limitations; they can be influenced by volume and correlate poorly with clinical acceptability.^43^, ^44^ Moreover, considering that the manual contours used as the GT inherently contain inter‐observer variability, relying solely on quantitative metrics may not yield a comprehensive assessment. Therefore, a qualitative evaluation was performed by four independent reviewers: two experienced radiation oncologists and two medical physicists. The AI contours were visually reviewed and scored on a 5‐point scale based on the criteria outlined in Table 2. A score of 3 was defined as the minimum threshold for clinical acceptability. An organ was classified as a “Failure” if two or more evaluators assigned a score of 2 or lower (i.e., judged the AI segmentation to be clinically unacceptable). Conversely, any organ not classified as a “Failure” was defined as a “Success.”

**TABLE 2:** Qualitative evaluation criteria

Score	Criteria
5	**Acceptable**: clinically usable without modification
4	**Acceptable**: clinically usable despite minor deviations
3	**Acceptable**: requires revisions for clinical use
2	**Unacceptable**: requires complete redrawing
1	**Unacceptable**: organ not recognized

Furthermore, to identify the causes of the failed organs, the contours were visually evaluated and categorized by an experienced medical physicist. Based on previous reports, the causes of failure were classified into four categories: setup position, anatomy, image artifacts, and unknown.^28^

Statistical  analyses were performed to compare the quantitative metrics (DSC and HD95) between the typical and atypical cohorts. Because segmentation evaluation metrics generally do not follow a normal distribution, the non‐parametric Mann‐Whitney U test was employed. Due to the small sample sizes of the specific atypical subgroups, statistical testing was not performed for individual subgroups; instead, the analysis was restricted to a two‐group comparison between the overall typical and atypical cohorts (both overall and per‐organ). All statistical analyses were conducted using Python 3.12 with the SciPy library.

### 2.4 Dosimetric evaluation

In addition to quantitative and qualitative assessments, a dosimetric evaluation was conducted to further verify the clinical utility of the AI auto‐segmentation. Because the thoracic dataset exhibited variations in dose fractionation and treatment strategies within the cohort, making homogeneous comparisons difficult, this evaluation was restricted to the male pelvic region (n = 34). Forward dosimetric analysis was employed for this evaluation. All dose distributions were optimized for a prescribed dose of 62 Gy in 20 fractions by experienced medical physicists using either Planning Station (Accuray Inc., Sunnyvale, CA, USA), RayStation, or Pinnacle treatment planning systems, and were actually delivered to the patients. The dose calculation grid size was 2 mm. Without re‐optimizing the dose distributions for this study, the existing dose distributions were directly applied to both the GT and AI contours to calculate and compare the dose‐volume metrics for the rectum and bladder. The specific dose‐volume constraints evaluated were those actually used in our clinical practice: D2% < 61 Gy, D10% < 55 Gy, and D50% < 18 Gy for the rectum, and D15% < 60 Gy, D25% < 58 Gy, D35% < 56 Gy, and D50% < 48 Gy for the bladder.

## 3 RESULTS

Table 3 summarizes the mean (± standard deviation) auto‐segmentation time and the normalized time per structure for each anatomical region. The mean auto‐segmentation time was 86.0 ± 13.9 sec for the thorax and 90.3 ± 12.1 sec for the male pelvis. Furthermore, the normalized time per structure was 10.8 ± 1.7 sec/structure for the thorax and 15.5 ± 2.0 sec/structure for the male pelvis.

**TABLE 3:** Mean auto‐segmentation time and normalized time per structure by anatomical region

	Thorax	Male pelvis
Auto‐segmentation time [sec]	86.0 ± 13.9	90.3 ± 12.1
Time per structure [sec/structure]	10.8 ± 1.7	15.5 ± 2.0
		(Ave ± SD)

**FIGURE 1:**
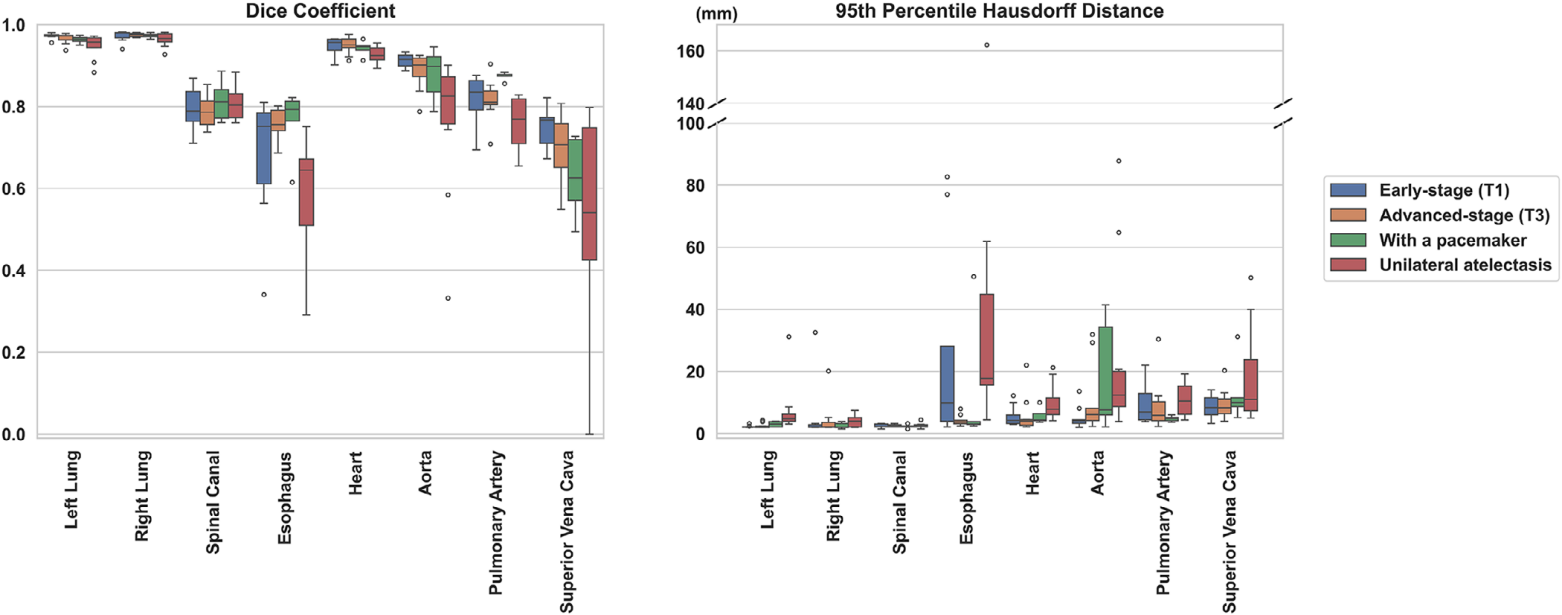
Boxplots of the Dice similarity coefficient and 95th percentile Hausdorff distance for typical and atypical cohorts in the thoracic region.

Figure 1 displays boxplots of the DSC and HD95 across all organs in the thoracic region for each cohort. Although the typical cohort achieved high accuracy (mean DSC: 0.856 ± 0.113; mean HD95: 6.89 ± 10.25 mm), performance trended lower in the atypical cohorts (mean DSC: 0.808 ± 0.176, *p =* 0.0457; mean HD95: 12.22 ± 19.96 mm, *p =* 0.0002). Specifically, results across the subgroups were as follows: the early‐stage (T1) cohort (mean DSC: 0.858 ± 0.119; mean HD95: 7.76 ± 13.04 mm), the advanced‐stage (T3) cohort (mean DSC: 0.854 ± 0.107; mean HD95: 6.02 ± 6.37 mm), the cohort with a pacemaker (mean DSC: 0.854 ± 0.122; mean HD95: 7.81 ± 11.18 mm), and the unilateral atelectasis cohort (mean DSC: 0.784 ± 0.194; mean HD95: 14.46 ± 22.92 mm).

**FIGURE 2:**
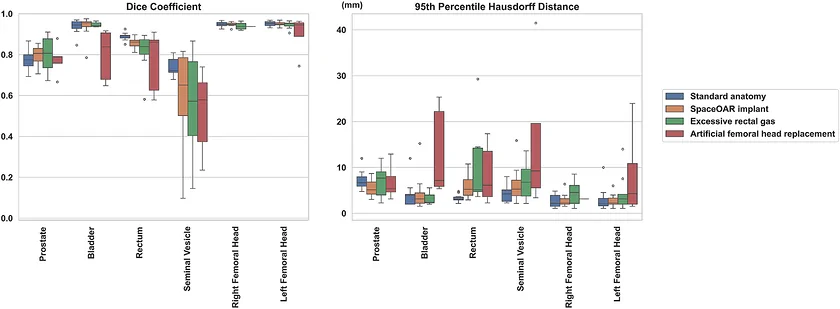
Boxplots of the Dice similarity coefficient and 95th percentile Hausdorff distance for typical and atypical cohorts in the male pelvic region.

A similar trend was observed in the male pelvic region, as illustrated in Figure 2. When compared with the typical cohort, the atypical cohorts showed a decrease in overall volumetric overlap (mean DSC: 0.828 ± 0.168, *p =* 0.1620) alongside a degradation in boundary errors (mean HD95: 6.16 ± 5.79 mm, *p =* 0.0075). A closer look at the subgroups revealed higher accuracy for the standard anatomy cohort (mean DSC: 0.874 ± 0.091; mean HD95: 4.11 ± 2.53 mm). In contrast, results were lower for the SpaceOAR implant cohort (mean DSC: 0.850 ± 0.153; mean HD95: 4.65 ± 3.05 mm), the excessive rectal gas cohort (mean DSC: 0.838 ± 0.169; mean HD95: 5.89 ± 4.84 mm), and particularly the artificial femoral head replacement cohort, which showed the most substantial decrease (mean DSC: 0.754 ± 0.183; mean HD95: 10.38 ± 9.69 mm).

**FIGURE 3:**
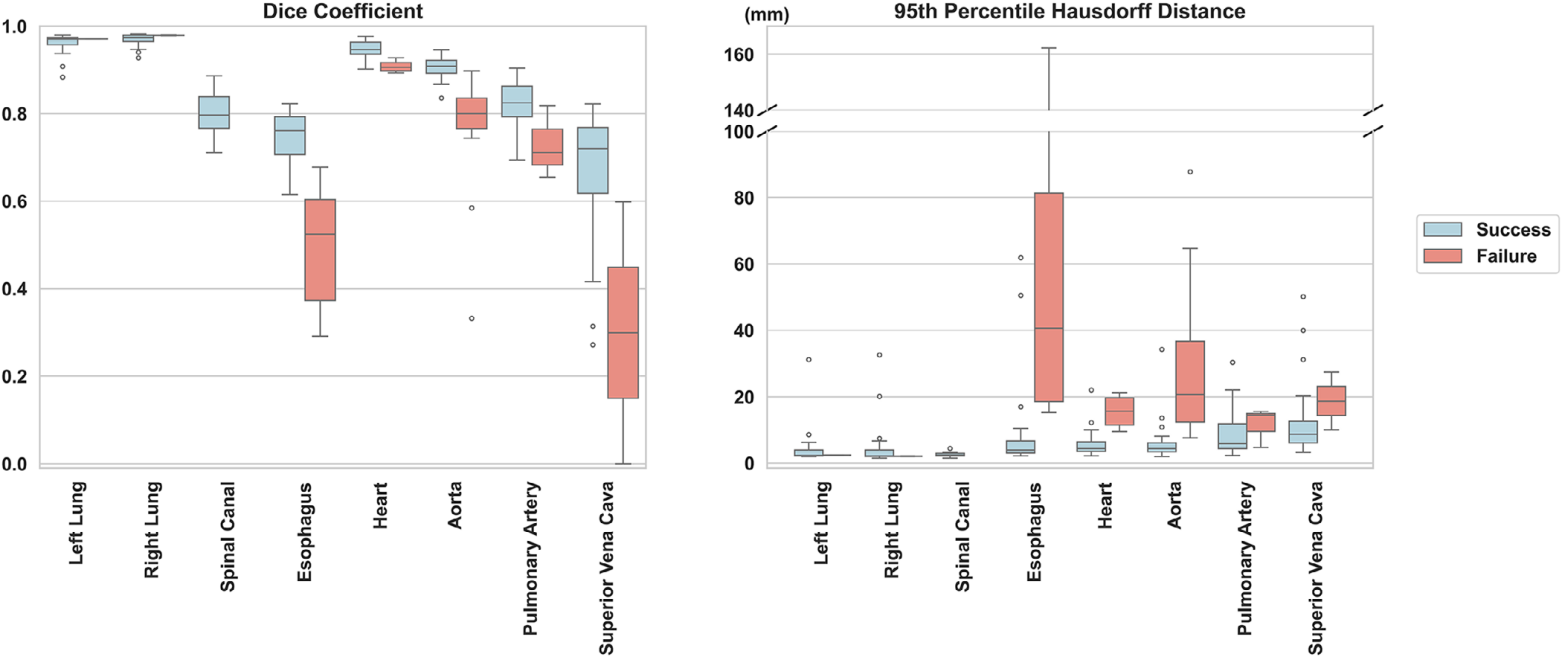
Boxplots of the Dice similarity coefficient and 95th percentile Hausdorff distance stratified by visual assessment results in the thoracic region. In this study, an organ was classified as a “Failure” when two or more evaluators assigned a score of 2 or lower.

**FIGURE 4:**
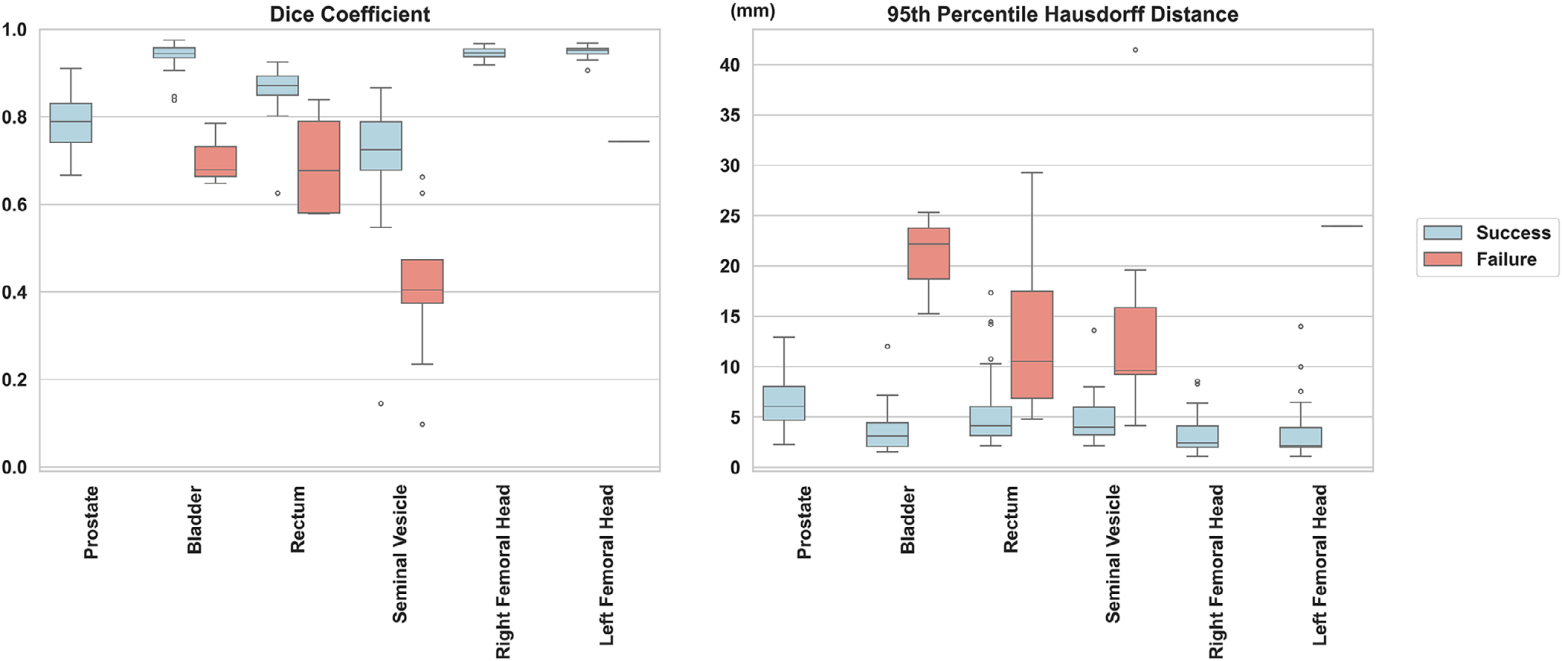
Boxplots of the Dice similarity coefficient and 95th percentile Hausdorff distance stratified by visual assessment results in the male pelvic region.

Figures 3 and 4 show boxplots of the DSC for cases visually assessed as “Success” or “Failure.” Table 4 presents the proportion of cases with a DSC exceeding 0.8 and the failure rate for each organ. More detailed failure rates for each cohort are presented in Supplementary Tables S1 and S2. Overall, accuracy tended to decrease in atypical cases, although there were exceptions, such as one typical case involving the left lung that was classified as a “Failure.” Notably, visual assessment did not always align with high DSC values; for instance, although all prostate cases were assessed as “Success,” the proportion of cases with a DSC greater than 0.8 was only 41.2%.

**TABLE 4:** Proportion of cases with a Dice similarity coefficient > 0.8 and failure rates based on visual assessment for each organ

Region	Organ	DSC > 0.8 (%)	Total Failure Rate (%)	Typical Cases Failure Rate (%)	Atypical Cases Failure Rate (%)
Thorax	Left lung	100.0	2.9	5.0	0.0
	Right lung	100.0	5.7	0.0	13.3
	Spinal canal	42.9	0.0	0.0	0.0
	Esophagus	14.7	26.5	15.8	40.0
	Heart	100.0	11.4	0.0	26.7
	Aorta	85.7	31.4	10.0	60.0
	Pulmonary artery	65.7	8.6	0.0	20.0
	Superior vena cava	8.6	5.7	0.0	13.3
Male Pelvis	Prostate	41.2	0.0	0.0	0.0
	Bladder	91.2	8.8	0.0	12.5
	Rectum	88.2	11.8	0.0	16.7
	Seminal vesicle	17.6	26.5	0.0	37.5
	Right femoral head	100.0	0.0	0.0	0.0
	Left femoral head	97.0	2.9	0.0	4.2

Table 5 categorizes the reasons for failure in the cases classified as “Failure.” No failures were attributed to setup position. The most common cause of failure was anatomical factors, followed by artifacts caused by foreign bodies. Additionally, errors with unidentified causes were observed in two esophagus cases and two seminal vesicle cases.

**TABLE 5:** Reasons for failure in cases classified as "Failure"

Region	Organ	Total failures	Failure reason:	Setup position	Anatomy	Image artifacts	Unknown
Thorax	Left lung	1		0	1	0	0
	Right lung	2		0	2	0	0
	Spinal canal	0		0	0	0	0
	Esophagus	9		0	6	1	2
	Heart	4		0	4	0	0
	Aorta	11		0	11	0	0
	Pulmonary artery	3		0	3	0	0
	Superior vena cava	2		0	2	0	0
Male Pelvis	Prostate	0		0	0	0	0
	Bladder	3		0	1	2	0
	Rectum	4		0	1	3	0
	Seminal vesicle	9		0	4	3	2
	Right femoral head	0		0	0	0	0
	Left femoral head	1		0	0	1	0

### 3.1 Dosimetric evaluation

Table 6 summarizes the dosimetric comparison results between the GT and AI contours for the bladder and rectum. Overall, the dose metrics based on AI contours tended to be slightly higher than those based on GT (mean dose difference: +0.01 to +2.33 Gy). Cases where the dose constraint shifted from “Pass” with GT to “Fail” with AI contours were observed in the bladder (5 cases) and rectum (7 cases), with individual cases affecting multiple constraints. In contrast, a shift from “Fail” to “Pass” was observed in only one case for the rectum D2%, which notably involved a substantial dose underestimation of ‐4.25 Gy by the AI contour. Importantly, among the cases where the constraint status shifted in the bladder, one case each for D15% and D25% had already been judged as “Failure” in the prior visual assessment.

**TABLE 6:** Dosimetric comparison results between the manual and AI contours for the bladder and rectum

Organ	Dose Constraint	Δdose (AI ‐ Manual) (Gy)	Pass → Fail	Fail → Pass	Remarks
Bladder	D15% < 60 Gy	+1.43 ± 3.95	5	0	One Pass‐to‐Fail case was visually judged as Failure.
	D25% < 58 Gy	+1.78 ± 5.21	1	0	One Pass‐to‐Fail case was visually judged as Failure.
	D35% < 56 Gy	+1.10 ± 5.20	0	0	
	D50% < 48 Gy	+0.78 ± 3.31	0	0	
Rectum	D2% < 61 Gy	+2.33 ± 4.11	6	1	
	D10% < 55 Gy	+1.29 ± 4.75	1	0	
	D50% < 18 Gy	+0.01 ± 1.87	1	0	

## 4 DISCUSSION

AI‐based automated segmentation is increasingly integrated into clinical practice to standardize delineation. In this study, we evaluated the accuracy of RatoGuide, a newly released deep learning‐based auto‐segmentation software system, using a dataset encompassing clinical cases derived from routine practice. Unlike previous studies that relied solely on general geometric metrics, our study employed a comprehensive approach, incorporating stratified comparisons of typical and atypical cases alongside expert visual assessments across both thoracic and pelvic regions. Our findings demonstrated that while typical cases maintained high segmentation accuracy (mean DSC: 0.856 and mean HD95: 6.89 mm for the thoracic region, and 0.874 and 4.11 mm for the male pelvic region), performance was notably compromised in atypical cases with anatomical variations or artifacts. Specifically, we observed a significant decrease in overall accuracy for the atypical cohorts within the thoracic region (mean DSC: 0.808; mean HD95: 12.22 mm). In the male pelvic region, while the reduction in volumetric overlap did not reach statistical significance, the atypical cohorts showed a clear downward trend (mean DSC: 0.828) coupled with significantly worsened boundary errors (mean HD95: 6.16 mm). Crucially, this study evaluated challenging scenarios, such as unilateral atelectasis and artificial femoral head replacements, where the mean DSCs dropped substantially to 0.784 and 0.754, and the mean HD95s worsened to 14.46 mm and 10.38 mm, respectively. These findings strongly highlight the necessity of evaluating software performance using atypical clinical cases.^27^ On the other hand, it is noteworthy that the software achieved these accuracy levels despite such real‐world complexities. The auto‐segmentation accuracy of RatoGuide is broadly consistent with previously published data. Doolan et al. evaluated five commercial auto‐segmentation software packages and reported median volumetric DSCs ranging from 0.82 to 0.88.^12^ For comparison, the median DSC for RatoGuide in this study was 0.878. However, while studies such as Doolan et al. primarily reported overall geometric accuracy, our stratified analysis and visual evaluations provide novel insights into specific failure modes in edge cases that general metrics alone fail to capture. Although a direct comparison is challenging due to differences in patient cohorts and target organs, these results fall within the range of previously reported values, validating the software's baseline performance while highlighting the added value of our comprehensive evaluation methodology.

An important finding from the comparison between the typical and atypical cohorts is that the impact of atypical factors on segmentation accuracy was not uniform but rather exhibited complex, system‐specific variations. For instance, the degradation in the accuracy of thoracic organs (lungs, heart, and blood vessels) due to atelectasis and the increased errors in the seminal vesicles and rectum following SpaceOAR implantation, were expected outcomes resulting from significant anatomical distortion. Conversely, in cases with implanted pacemakers, neither a decrease in cardiac segmentation accuracy nor any “Failure” classification was observed despite the presence of metal artifacts, strongly suggesting that such cases were adequately represented in the training dataset (Figure S1). Furthermore, even within the typical cohort lacking anatomical deformations, the esophagus exhibited a high failure rate of 15.8%. This indicates an inherent difficulty in delineating the esophagus on non‐contrast CT due to its low soft‐tissue contrast, tortuous path, and susceptibility to compression.^13^, ^19^ These discrepancies in vulnerability to specific pathological conditions and performance stability against certain artifacts directly stem from the distribution of the model's training data and its learning architecture—details that are rarely disclosed by manufacturers.^28^ Therefore, to fully understand the specific limitations and failure patterns of these black‐box commercial software tools, it is essential for users to intentionally prepare typical and atypical clinical datasets and systematically evaluate the software within their own institutions.^27^

Combining multiple metrics is essential for evaluating segmentation accuracy. For example, in the thoracic region, the mean DSC showed favorable values of 0.856 for the typical cohort and 0.808 for the atypical cohort. Conversely, the HD95 was 6.89 mm and 12.22 mm, respectively, indicating “poor agreement” even in the typical cohort.^12^ In this study, the primary cause of this HD95 degradation was the poor performance in delineating the esophagus. In some cases, DL‐generated esophageal contours were observed to terminate abruptly along the longitudinal axis (Figure S2). In such instances, the impact on overall volumetric overlap (DSC) is limited, but HD95, which reflects the maximum boundary error, worsens significantly. Because DSC and HD95 evaluate different aspects of segmentation, a multifaceted evaluation is required for clinical implementation.^43^, ^44^

Furthermore, visual assessment provides a more clinically relevant perspective. In this study, discrepancies were observed between quantitative metrics and visual assessments. For instance, some esophageal and bladder cases with poor quantitative metrics were judged as “Success,” whereas in one case with atelectasis, the lung was rated as “Failure” despite favorable quantitative metrics. This occurs because auto‐segmentation is intended to provide initial contours for manual editing rather than final products. The clinical utility of auto‐segmentation for organs at risk depends more on the ease of manual correction than on pure geometric accuracy.^45^ This discrepancy is illustrated in the relationship between organ volume and accuracy (Figures S3, S4). Small‐volume OARs tended to exhibit lower DSCs and more “Failure” ratings. While lower DSCs are largely due to the mathematical property of the metric itself, where minor boundary errors disproportionately penalize the score in small structures, correcting these contours typically requires minimal manual intervention. Systematic errors, such as missing slices at the borders or uniformly small contours, are straightforward to correct and thus clinically acceptable.^46^ The esophageal cases in our study exemplify this dichotomy in error types: while severely irregular boundaries led to a “Failure” rating (as seen in 15.8% of the typical cohort), cases with abrupt longitudinal termination (missing slices) resulted in lower quantitative accuracy but were rated as “Success” because adding a few contours requires only minor adjustments. Conversely, contours with irregular boundaries require extensive slice‐by‐slice modification and are considered a “Failure” even if the overall DSC is high.

In contrast, this “editing complexity” rationale does not apply to treatment targets such as the prostate and seminal vesicles. This is because contour deviations in target volumes lead to inadequate radiation dosing to the tumor, directly impacting clinical outcomes. Although all prostate cases were visually assessed as “Success,” only 41.2% achieved a DSC greater than 0.8. As for the seminal vesicles, while 73.5% were rated as “Success,” a mere 17.6% achieved a DSC greater than 0.8. In this study, a “Success” rating merely indicates from a qualitative perspective that there are no fatal misidentifications and that the structures are viable as initial contours. As long as the DSC deviates from 1.0, rigorous slice‐by‐slice modification is required to ensure complete tumor coverage, regardless of the organ's volume. Given these differences in the evaluation requirements for OARs and targets, it remains crucially important to combine visual assessments that consider the complexity of manual modification with strict geometric validation to achieve a comprehensive evaluation.^16^

In our dosimetric evaluation, AI contours tended to yield higher dose metrics. This is likely because the AI was prone to underestimating the volumes of the rectum and bladder, leading to a relative increase in the proportion of high‐dose regions. Although previous studies have reported dosimetric discrepancies between GT and AI contours, their clinical impact is generally considered minor.^47^, ^48^ However, in this study, one case that should have failed the dose constraint was misjudged as a “Pass” when evaluated using the AI contour. This anomaly occurred when the AI failed to delineate the specific portion of the organ adjacent to the high‐dose target volume, resulting in a substantial underestimation of the actual dose (by 4.25 Gy in this instance). This represents a critical false‐negative error that overlooks excessive dosing, necessitating particular caution in clinical implementation. Nevertheless, due to variations in treatment strategies, the dosimetric evaluation in this study was restricted to specific anatomical regions, and further investigations using larger datasets are required.

This study has several limitations. First, as a single‐vendor study, our evaluation focused exclusively on RatoGuide without direct comparison to other commercial solutions, meaning our findings should not be overgeneralized. Future research should aim to perform a comparative analysis using multiple software packages to ensure a more robust evaluation. Second, the patient cohort was small and limited to a single institution. Since institution‐specific parameters, such as CT acquisition protocols, can influence auto‐segmentation accuracy, larger‐scale, multi‐institutional studies are necessary to validate these findings. Third, the GT contours used in this study were derived from clinical practice and thus contain inherent inter‐observer variability. While reviewed by experts, it should be acknowledged that the GT itself may not be the absolute gold standard. Furthermore, the lack of an inter‐observer baseline comparison makes it difficult to determine whether the AI's geometric errors fall within acceptable human variation. Fourth, the absence of uncertainty estimation limits the ability to quantify confidence levels for AI predictions. Finally, due to the retrospective design, we could not measure manual contouring or editing times. Thus, no conclusions can be drawn regarding actual time savings or workload reduction. Previous reports have highlighted the importance of reporting absolute and relative time savings; therefore, future prospective studies are necessary for these analyses.^17^, ^43^

## 5 CONCLUSION

RatoGuide demonstrated favorable performance in typical cases, though accuracy declined in atypical cases with artifacts or altered anatomy. For clinical implementation, clinicians should prioritize the manual review of atypical cases and organs in high‐dose gradient regions, where minor geometric errors can alter dosimetric compliance. Future work should include prospective multi‐institutional validations and large‐scale comparative dosimetric evaluations involving multiple commercial solutions.

## AUTHOR CONTRIBUTIONS

Ryota Tozuka and Noriyuki Kadoya contributed to the conception and design of this study. Masahide Saito, Masaki Matsuda, Tomoko Akita, Hikaru Nemoto, Zhe Chen, and Kazuma Mochizuki conducted the primary analysis. Ryota Tozuka mainly drafted the manuscript. Noriyuki Kadoya, Takafumi Komiyama, Keiichi Jingu, and Hiroshi Onishi reviewed the manuscript. All authors read and approved the final manuscript.

## CONFLICT OF INTEREST STATEMENT

Noriyuki Kadoya serves as a board member of AiRato Inc. Both Noriyuki Kadoya and Ryota Tozuka hold stock in the company, while Hiroshi Onishi and Keiichi Jingu received grants from AiRato Inc.

## ETHICAL APPROVAL

This study was approved by the institutional review board of the University of Yamanashi. The approval number was CS0010.

## Supporting information



Supporting Information

## Data Availability

Authors will share data upon reasonable request to the corresponding author.

## References

[1] Li XA , Tai A , Arthur DW , et al. Variability of target and normal structure delineation for breast cancer radiotherapy: an RTOG Multi‐Institutional and Multiobserver Study. Int J Radiat Oncol Biol Phys. 2009;73(3):944‐951. doi:10.1016/j.ijrobp.2008.10.034 19215827 PMC2911777

[2] Vinod SK , Jameson MG , Min M , Holloway LC . Uncertainties in volume delineation in radiation oncology: a systematic review and recommendations for future studies. Radiother Oncol. 2016;121(2):169‐179. doi:10.1016/j.radonc.2016.09.009 27729166

[3] Nelms BE , Tomé WA , Robinson G , Wheeler J . Variations in the contouring of organs at risk: test case from a patient with oropharyngeal cancer. Int J Radiat Oncol Biol Phys. 2012;82(1):368‐378. doi:10.1016/j.ijrobp.2010.10.019 21123004

[4] Jaikuna T , Osorio EV , Azria D , et al. Contouring variation affects estimates of normal tissue complication probability for breast fibrosis after radiotherapy. Breast. 2023;72:103578. doi:10.1016/j.breast.2023.103578 37713940 PMC10511799

[5] Marin T , Zhuo Y , Lahoud RM , et al. Deep learning‐based GTV contouring modeling inter‐ and intra‐ observer variability in sarcomas. Radiother Oncol. 2022;167:269‐276. doi:10.1016/j.radonc.2021.09.034 34808228 PMC8934266

[6] Erdur AC , Rusche D , Scholz D , et al. Deep learning for autosegmentation for radiotherapy treatment planning: state‐of‐the‐art and novel perspectives. Strahlenther Onkol. 2025;201(3):236‐254. doi:10.1007/s00066-024-02262-2 39105745 PMC11839850

[7] Tozuka R , Kadoya N , Yasunaga A , et al. Fractal‐driven self‐supervised learning enhances early‐stage lung cancer GTV segmentation: a novel transfer learning framework. Jpn J Radiol. 2026;44(1):183‐195. doi:10.1007/s11604-025-01865-8 40952548 PMC12769625

[8] Ginn JS , Gay HA , Hilliard J , et al. A clinical and time savings evaluation of a deep learning automatic contouring algorithm. Med Dosim. 2023;48(1):55‐60. doi:10.1016/j.meddos.2022.11.001 36550000

[9] Han Z , Wang Y , Wang W , et al. Artificial intelligence‐assisted delineation for postoperative radiotherapy in patients with lung cancer: a prospective, multi‐center, cohort study. Front Oncol. 2024;14:1388297. doi:10.3389/fonc.2024.1388297 39575415 PMC11579590

[10] Choi MS , Chang JS , Kim K , et al. Assessment of deep learning‐based auto‐contouring on interobserver consistency in target volume and organs‐at‐risk delineation for breast cancer: implications for RTQA program in a multi‐institutional study. Breast. 2024;73:103599. doi:10.1016/j.breast.2023.103599 37992527 PMC10700624

[11] Pang EPP , Tan HQ , Wang F , et al. Multicentre evaluation of deep learning CT autosegmentation of the head and neck region for radiotherapy. NPJ Digit Med. 2025;8(1):312. doi:10.1038/s41746-025-01624-z 40419731 PMC12106707

[12] Doolan PJ , Charalambous S , Roussakis Y , et al. A clinical evaluation of the performance of five commercial artificial intelligence contouring systems for radiotherapy. Front Oncol. 2023;13:1213068. doi:10.3389/fonc.2023.1213068 37601695 PMC10436522

[13] Kim YW , Biggs S , Claridge Mackonis E . Investigation on performance of multiple AI‐based auto‐contouring systems in organs at risks (OARs) delineation. Phys Eng Sci Med. 2024;47(3):1123‐1140. doi:10.1007/s13246-024-01434-9 39222214 PMC11408550

[14] Meyer C , Huger S , Bruand M , et al. Artificial intelligence contouring in radiotherapy for organs‐at‐risk and lymph node areas. Radiat Oncol. 2024;19(1):168. doi:10.1186/s13014-024-02554-y 39574153 PMC11580215

[15] Choi SH , Park JW , Cho Y , Yang G , Yoon HI . Automated Organ Segmentation for Radiation Therapy: a Comparative Analysis of AI‐Based Tools Versus Manual Contouring in Korean Cancer Patients. Cancers (Basel). 2024;16(21):3670.doi:10.3390/cancers16213670 39518109 PMC11544936

[16] Rong Y , Chen Q , Fu Y , et al. NRG Oncology Assessment of Artificial Intelligence Deep Learning‐Based Auto‐segmentation for Radiation Therapy: current Developments, Clinical Considerations, and Future Directions. Int J Radiat Oncol Biol Phys. 2024;119(1):261‐280. doi:10.1016/j.ijrobp.2023.10.033 37972715 PMC11023777

[17] Rusanov B , Ebert MA , Sabet M , et al. Guidance on selecting and evaluating AI auto‐segmentation systems in clinical radiotherapy: insights from a six‐vendor analysis. Phys Eng Sci Med. 2025;48(1):301‐316. doi:10.1007/s13246-024-01513-x 39804550 PMC11997002

[18] Fan M , Wang T , Lei Y , et al. Evaluation and failure analysis of four commercial deep learning‐based autosegmentation software for abdominal organs at risk. J Appl Clin Med Phys. 2025;26(4):e70010. doi:10.1002/acm2.70010 39946266 PMC11969109

[19] Yuan L , Chen Q , Al‐Hallaq H , et al. Quantitative Evaluation of Artificial Intelligence‐Based Organ Segmentation Across Multiple Anatomic Sites Using 8 Commercial Software Platforms. Pract Radiat Oncol. 2026;16(1):e47‐e59. doi:10.1016/j.prro.2025.06.012 40854402 PMC12794587

[20] Cardenas CE , Yang J , Anderson BM , Court LE , Brock KB . Advances in Auto‐Segmentation. Semin Radiat Oncol. 2019;29(3):185‐197. doi:10.1016/j.semradonc.2019.02.001 31027636

[21] Vandewinckele L , Claessens M , Dinkla A , et al. Overview of artificial intelligence‐based applications in radiotherapy: recommendations for implementation and quality assurance. Radiother Oncol. 2020;153:55‐66. doi:10.1016/j.radonc.2020.09.008 32920005

[22] Kalantar R , Lin G , Winfield JM , et al. Automatic Segmentation of Pelvic Cancers Using Deep Learning: state‐of‐the‐Art Approaches and Challenges. Diagnostics (Basel). 2021;11(11):1964. doi:10.3390/diagnostics11111964 34829310 PMC8625809

[23] Isaksson LJ , Summers P , Mastroleo F , et al. Automatic Segmentation with Deep Learning in Radiotherapy. Cancers (Basel). 2023;15(17):4389. doi:10.3390/cancers15174389 37686665 PMC10486603

[24] Bibault JE , Giraud P . Deep learning for automated segmentation in radiotherapy: a narrative review. Br J Radiol. 2024;97:13‐20. doi:10.1093/bjr/tqad018 38263838 PMC11027240

[25] Ng CK . Performance of commercial deep learning‐based auto‐segmentation software for prostate cancer radiation therapy planning: a systematic review. Information. 2025;16(3):215. doi: 10.3390/info16030215

[26] Kelly CJ , Karthikesalingam A , Suleyman M , Corrado G , King D . Key challenges for delivering clinical impact with artificial intelligence. BMC Med. 2019;17(1):195. doi:10.1186/s12916-019-1426-2 31665002 PMC6821018

[27] Mackay K , Banfill K , Bernstein D , et al. Royal College of Radiologists Guidance Statements on the Use of Auto‐contouring in Radiotherapy. Clin Oncol (R Coll Radiol). 2026;50:104004. doi:10.1016/j.clon.2025.104004 41519097

[28] Temple SWP , Rowbottom CG . Gross failure rates and failure modes for a commercial AI‐based auto‐segmentation algorithm in head and neck cancer patients. J Appl Clin Med Phys. 2024;25(6):e14273. doi:10.1002/acm2.14273 38263866 PMC11163497

[29] Endo S , Nakamura M , Fujisawa T , Hojo H , Tachibana H . Cross‐anatomical evaluation of a deep‐learning auto‐contouring system: qualitative, geometric, and dosimetric validation. J Appl Clin Med Phys. 2026;27(6):e70662. doi:10.1002/acm2.70662 42298779 PMC13269653

[30] Kadoya N , Kimura Y , Tozuka R , et al. Evaluation of deep learning‐based deliverable VMAT plan generated by prototype software for automated planning for prostate cancer patients. J Radiat Res. 2023;64(5):842‐849. doi:10.1093/jrr/rrad058 37607667 PMC10516733

[31] Nemoto H , Saito M , Kadoya N , et al. Evaluation of deep learning‐based automated radiotherapy planning for early‐stage lung cancer using SBRT‐VMAT: a comparison with manual planning. J Appl Clin Med Phys. 2025;26(10):e70291. doi:10.1002/acm2.70291 41088571 PMC12521067

[32] Xiao Y , Tanaka S , Kadoya N , et al. Evaluation of deliverable artificial intelligence‐based automated volumetric arc radiation therapy planning for whole pelvic radiation in gynecologic cancer. Sci Rep. 2025;15(1):15219. doi:10.1038/s41598-025-99717-y 40307456 PMC12043927

[33] Nakajima T , Kadoya N , Tozuka R , et al. Evaluation of deliverable dose‐mimicking automated volumetric arc radiation therapy planning for stage III non‐small cell lung cancer patients: comparison with a commercial DVH‐predicted automated planning system. J Radiat Res. 2026;67(2):248‐258. doi:10.1093/jrr/rrag001 41642718 PMC13019132

[34] Nagake Y , Yasui K , Sugiyama A , et al. Clinical edge‐case stress testing of two Asia‐developed AI auto‐contouring systems using prostate radiotherapy planning CT images. Radiol Phys Technol. 2026. doi:10.1007/s12194-026-01080-8 42268569

[35] Kong FM , Ritter T , Quint DJ , et al. Consideration of dose limits for organs at risk of thoracic radiotherapy: atlas for lung, proximal bronchial tree, esophagus, spinal cord, ribs, and brachial plexus. Int J Radiat Oncol Biol Phys. 2011;81(5):1442‐1457. doi:10.1016/j.ijrobp.2010.07.1977 20934273 PMC3933280

[36] Feng M , Moran JM , Koelling T , et al. Development and validation of a heart atlas to study cardiac exposure to radiation following treatment for breast cancer. Int J Radiat Oncol Biol Phys. 2011;79(1):10‐18. doi:10.1016/j.ijrobp.2009.10.058 20421148 PMC2937165

[37] Gay HA , Barthold HJ , O'Meara E , et al. Pelvic normal tissue contouring guidelines for radiation therapy: a Radiation Therapy Oncology Group consensus panel atlas. Int J Radiat Oncol Biol Phys. 2012;83(3):e353‐62. doi:10.1016/j.ijrobp.2012.01.023 22483697 PMC3904368

[38] Jabbour SK , Hashem SA , Bosch W , et al. Upper abdominal normal organ contouring guidelines and atlas: a Radiation Therapy Oncology Group consensus. Pract Radiat Oncol. 2014;4(2):82‐89. doi:10.1016/j.prro.2013.06.004 24890348 PMC4285338

[39] Brouwer CL , Steenbakkers RJ , Bourhis J , et al. CT‐based delineation of organs at risk in the head and neck region: DAHANCA, EORTC, GORTEC, HKNPCSG, NCIC CTG, NCRI, NRG Oncology and TROG consensus guidelines. Radiother Oncol. 2015;117(1):83‐90. doi:10.1016/j.radonc.2015.07.041 26277855

[40] Finnegan RN , Quinn A , Horsley P , et al. Geometric and dosimetric evaluation of a commercial AI auto‐contouring tool on multiple anatomical sites in CT scans. J Appl Clin Med Phys. 2025;26(6):e70067. doi:10.1002/acm2.70067 40098297 PMC12148769

[41] Yu X , Yang Q , Zhou Y , et al. UNesT: local spatial representation learning with hierarchical transformer for efficient medical segmentation. Medical Image Analysis. 2023;90:102939. doi: 10.1016/j.media.2023.102939 37725868 10.1016/j.media.2023.102939 PMC11229077

[42] Brock KK , Mutic S , McNutt TR , Li H , Kessler ML . Use of image registration and fusion algorithms and techniques in radiotherapy: report of the AAPM Radiation Therapy Committee Task Group No. 132. Med Phys. 2017;44(7):e43‐e76. doi:10.1002/mp.12256 28376237

[43] Sherer MV , Lin D , Elguindi S , et al. Metrics to evaluate the performance of auto‐segmentation for radiation treatment planning: a critical review. Radiother Oncol. 2021;160:185‐191. doi:10.1016/j.radonc.2021.05.003 33984348 PMC9444281

[44] Mackay K , Bernstein D , Glocker B , Kamnitsas K , Taylor A . A review of the metrics used to assess auto‐contouring systems in radiotherapy. Clin Oncol (R Coll Radiol). 2023;35(6):354‐369. doi:10.1016/j.clon.2023.01.016 36803407

[45] Zhang Y , Amjad A , Ding J , et al. Comprehensive clinical usability‐oriented contour quality evaluation for deep learning auto‐segmentation: combining multiple quantitative metrics through machine learning. Pract Radiat Oncol. 2025;15(1):93‐102. doi:10.1016/j.prro.2024.07.007 39233005 PMC11711007

[46] Vaassen F , Hazelaar C , Vaniqui A , et al. Evaluation of measures for assessing time‐saving of automatic organ‐at‐risk segmentation in radiotherapy. Phys Imaging Radiat Oncol. 2020;13:1‐6. doi:10.1016/j.phro.2019.12.001 33458300 PMC7807544

[47] Kawula M , Purice D , Li M , et al. Dosimetric impact of deep learning‐based CT auto‐segmentation on radiation therapy treatment planning for prostate cancer. Radiat Oncol. 2022;17(1):21. doi:10.1186/s13014-022-01985-9 35101068 PMC8805311

[48] Podobnik G , Ibragimov B , Peterlin P , Strojan P , Vrtovec T . Dosimetric assessment of deep learning based organ‐at‐risk segmentation: insights from the HaN‐Seg challenge. Radiother Oncol. 2026;217:111387. doi:10.1016/j.radonc.2026.111387 41581701

