# Supplementary material for: Clinical evaluation of novel deep learning‐based auto‐segmentation software: Utility and potential pitfalls
Source: J Appl Clin Med Phys. 2026 Aug 25;27(9):e70757. doi: 10.1002/acm2.70757 (PMC13504373; doi:10.1002/acm2.70757)
Supplement: Supplementary file 1 [file ACM2-27-e70757-s001.pdf]

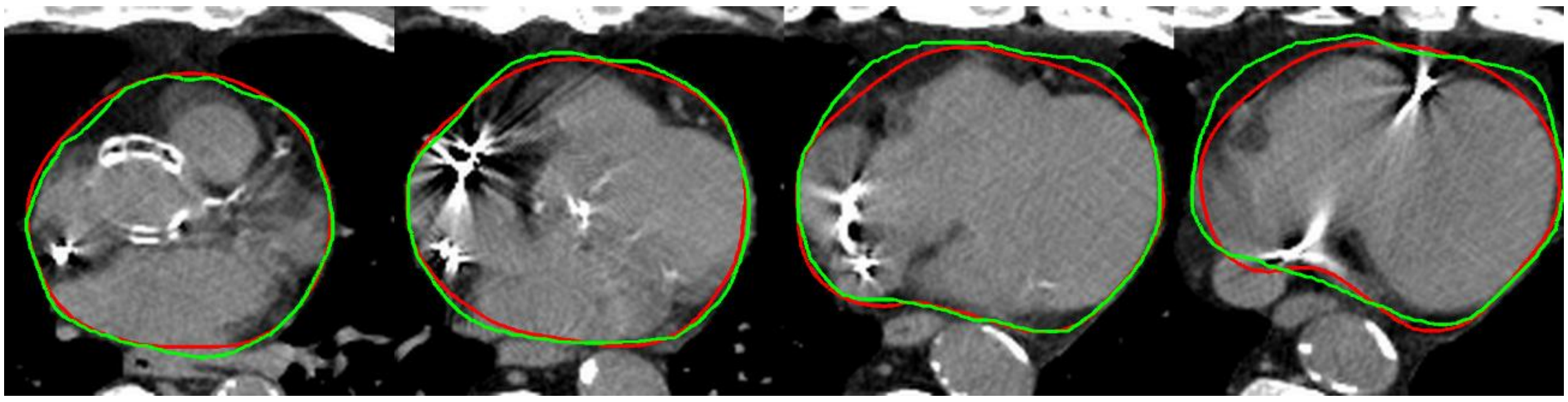

Supplementary Figure 1: Segmentation result in a patient with an implanted pacemaker. Despite metal artifacts caused by the pacemaker leads, the target structure was successfully delineated (green) relative to the ground truth (red).

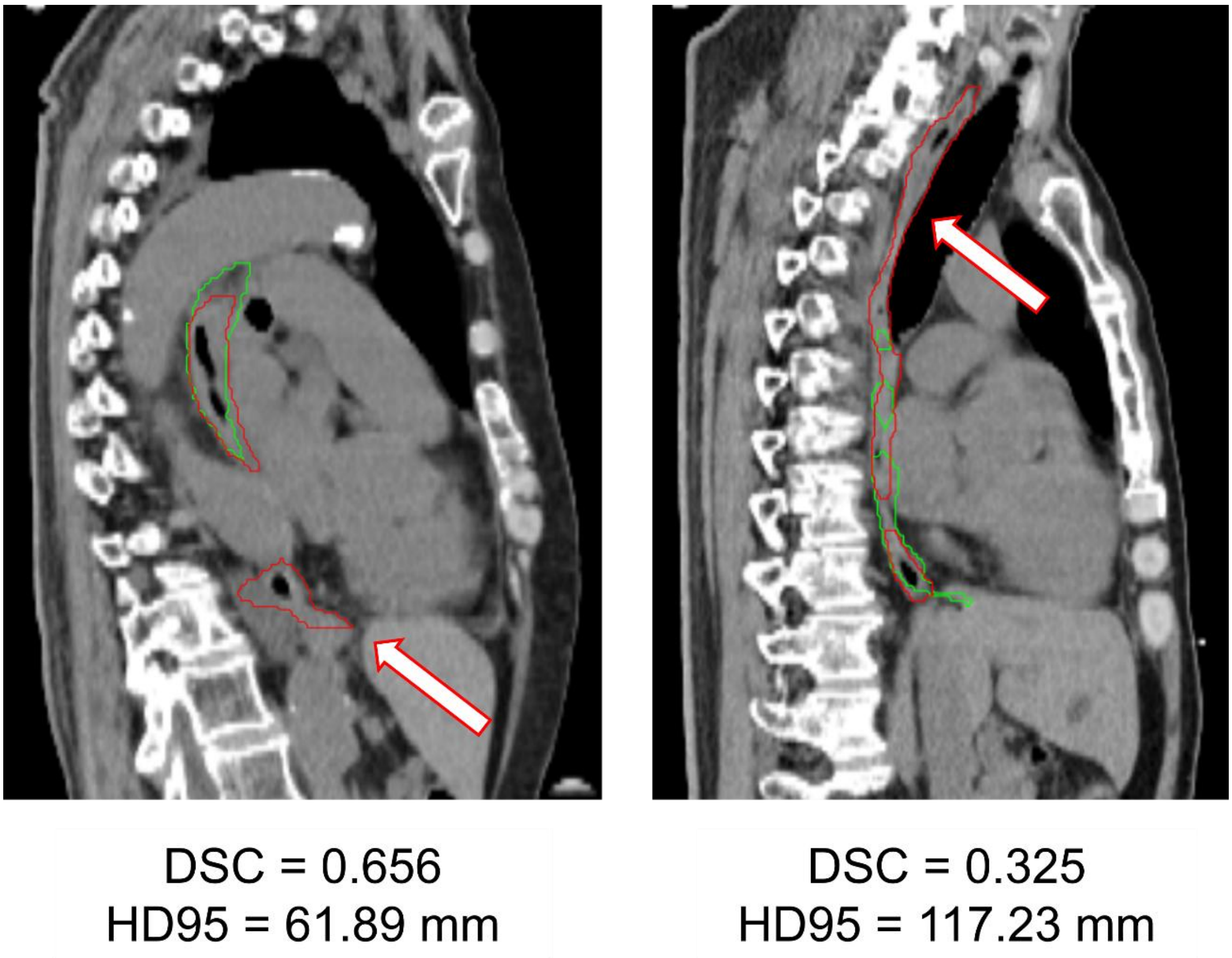

Supplementary Figure 2: Representative examples of an AI-generated esophageal contour terminating abruptly. In these cases, the Dice coefficient is preserved to some extent due to partial agreement, whereas the HD95 exhibits an outlier value.

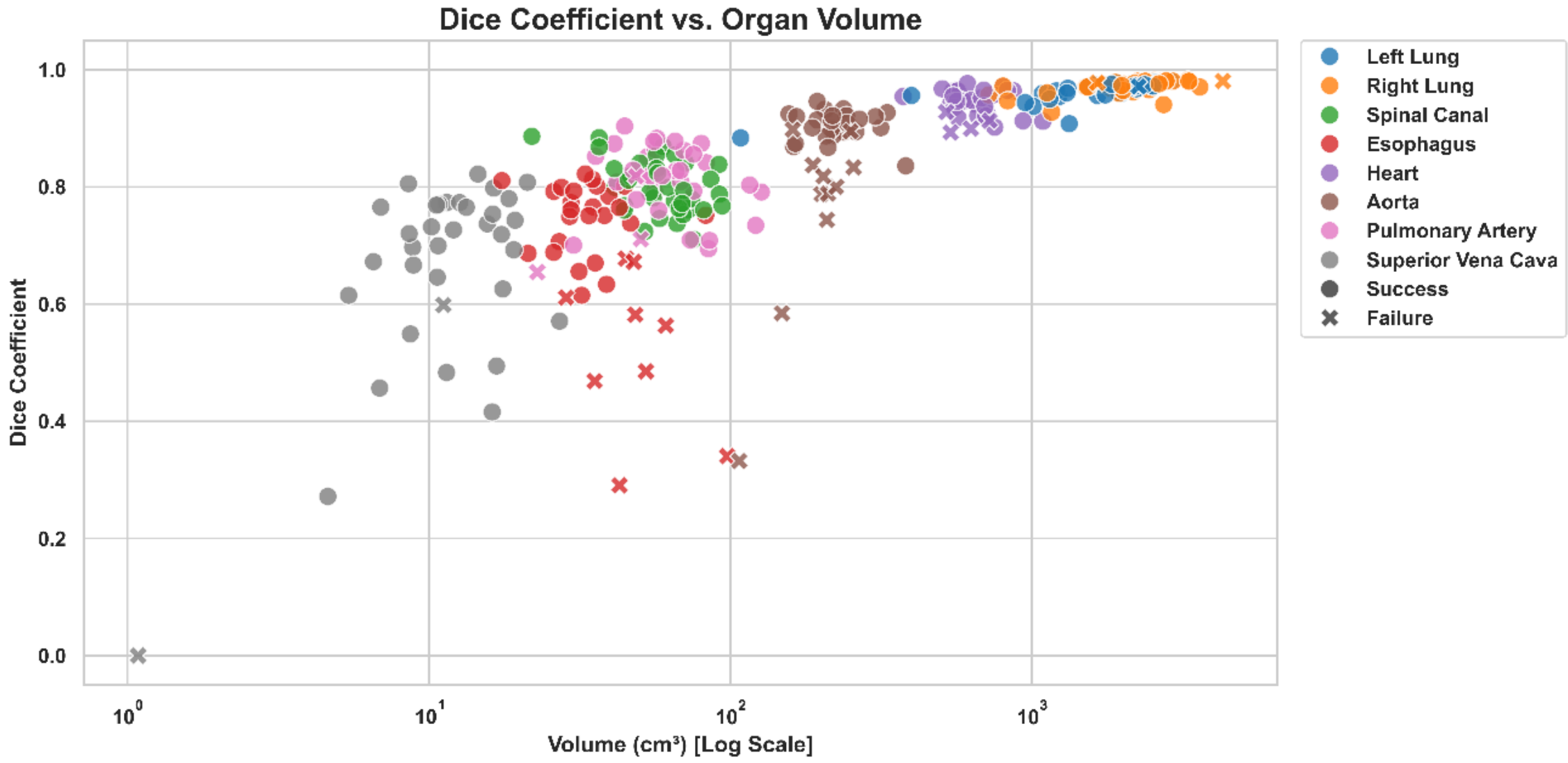

Supplementary Figure 3: Scatter plot of the Dice similarity coefficient (DSC) versus organ volume in the thorax region. The x-axis represents the organ volume on a logarithmic scale (cm^3), and the y-axis represents the DSC. Circles indicate cases visually assessed as Success, while crosses indicate Failure. This figure demonstrates that smaller structures (e.g., superior vena cava and esophagus) tend to yield lower DSCs and have a higher frequency of visual failures compared to larger organs (e.g., lungs and heart). In small-volume organs, even minor absolute geometric discrepancies can lead to significant relative errors, penalizing the DSC. Therefore, a lower DSC in these structures reflects the geometric sensitivity of the metric and does not necessarily equate to a proportional increase in the clinical editing burden.

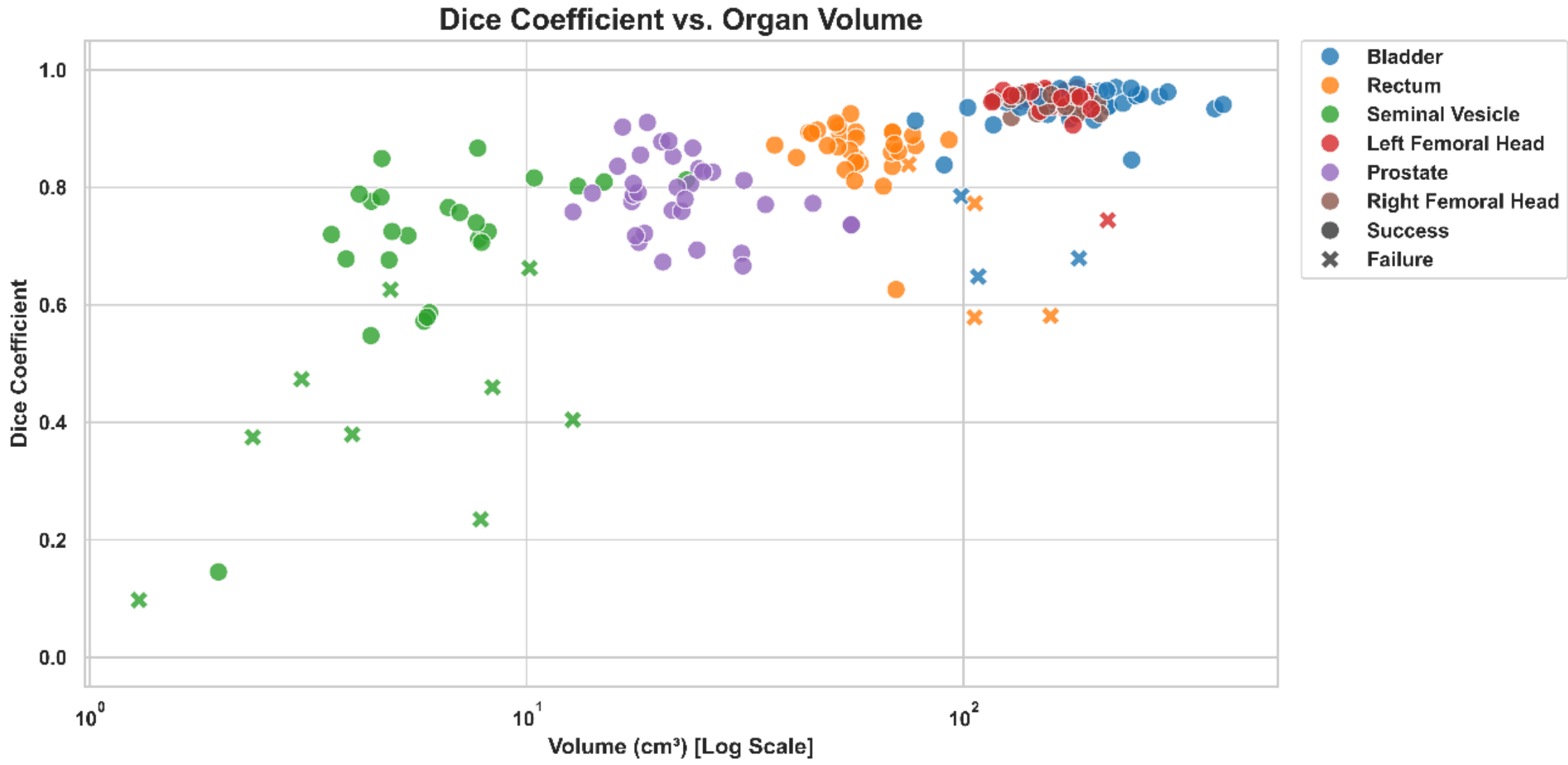

Supplementary Figure 4: Scatter plot of the Dice similarity coefficient (DSC) versus organ volume in the male pelvic region. While smaller structures (e.g., seminal vesicles) show lower DSCs and frequent visual failures, failures also occur in large organs (>100 cm³) like the rectum. This suggests that in the pelvis, the editing burden is influenced not only by geometric scale but also by organ-specific factors such as image artifacts and varying gas volumes.

Table S1. Total failure rate and failure rates across different cohorts for each organ in the thoracic region.

Organ	Total Failure Rate (%)	Failure Rate by Cohort (%)			
		Early-stage (T1)	Advanced-stage (T3)	With a pacemaker	Unilateral atelectasis
Left lung	2.9	10.0	0.0	0.0	0.0
Right lung	5.7	0.0	0.0	0.0	0.0
Spinal canal	0.0	0.0	0.0	0.0	0.0
Esophagus	26.5	33.3	0.0	0.0	60.0
Heart	11.4	0.0	0.0	0.0	40.0
Aorta	31.4	0.0	20.0	40.0	70.0
Pulmonary artery	8.6	0.0	0.0	0.0	30.0
Superior vena cava	5.7	0.0	0.0	0.0	20.0

Table S2. Total failure rate and failure rates across different cohorts for each organ in the male pelvic region

Organ	Total Failure Rate (%)	Failure Rate by Cohort (%)			
		Standard anatomy	SpaceOAR implant	Excessive rectal gas	Artificial femoral head replacement
Prostate	0.0	0.0	0.0	0.0	0.0
Bladder	8.8	0.0	10.0	0.0	40.0
Rectum	11.8	0.0	0.0	33.3	20.0
Seminal vesicle	26.5	0.0	40.0	22.2	60.0
Right femoral head	0.0	0.0	0.0	0.0	40.0
Left femoral head	2.9	0.0	0.0	0.0	20.0
